# Supplementary material for: Patterns of Intron Gain and Loss in Fungi
Source: PLoS Biol. 2004 Nov 30;2(12):e422. doi: 10.1371/journal.pbio.0020422 (PMC532390; doi:10.1371/journal.pbio.0020422)
Supplement: Table S1 — Also available at http://genes.mit.edu/NielsenEtAl/. (4.3 MB ZIP). [file pbio.0020422.st001.zip › NielsenEtAl/html/684.html]

AN8054.1.NCU06440.1.MG06472.1.FG07282.1


```
 CLUSTAL W (1.82) Multiple Sequence Alignments - Introns Inserted


Sequence 1: NCU06440.1	269 aa
Sequence 2: MG06472.1	269 aa
Sequence 3: FG07282.1	271 aa
Sequence 4: AN8054.1	269 aa
Alignment Length: 274 aa
Number Identitical Residues: 204 aa
Alignment Score (without introns) 8148


MG06472.1 	MASGYDRALS-V1FS~-~PDGHVFQVEYAGEAVKRG~TCAVGVKGADIVVLGCEKRSAMK
NCU06440.1	MASGYDRALS-V1FS2-~PDGHVFQVEYAGEAVKRG1TCAVGVKGKDVVVLGCEKRSAMK
FG07282.1 	MASGYDRALSGE~FP~Y2PDGHVFQVEYAGEAVKRG1TCAVGVKGTDVVVLGCEKRSAMK
AN8054.1  	-MSGYDRALS-V1FS2-~PDGHVFQVEYAMEAVKRG1TCAVGVKGKDVVVLGCEKRSALK
          	  ********   *.   *********** ****** ******** *:**********:*

MG06472.1 	LQDTRITPSKIGLIDTHVCLAFAGLNADARILVDKARLEAQSHRLTVEDPVTIEYITKYV
NCU06440.1	LQDTRITPSKIGLVDKHVCLAFAGLNADARILVDKARLEAQSHRLNLEDPVTIEYITKYV
FG07282.1 	LQDTRITPSKIQLLDHHVALAFAGLNADARILVDKARLEAQSHRLSVEDPVTIDYITKYV
AN8054.1  	LQDTRITPSKIAVLDNHAILAFAGLNADARILIDKARLEAQSHRLTVEDPVSIEYITKYI
          	*********** ::* *. *************:************.:****:*:*****:

MG06472.1 	AGVQQRYTQSGGVRPFGISTLVVGFDNGSKVPRLYQTEPSGIYSAW2KANAIGRSSKTVR
NCU06440.1	AGVQQRYTQSGGVRPFGISTLIVGFDNGSDVPRLYQTEPSGIYSAW2KANAIGRSSKTVR
FG07282.1 	AGVQQRYTQAGGVRPFGISTLIVGFDHGSDVPRLYQTEPSGIYSAW2KANAIGRSSKTVR
AN8054.1  	ATVQQRYTQSGGVRPFGISTLVVGFDPNDKTPRLYQTEPSGIYSAW2KANAIGRSSKTVR
          	* *******:***********:**** ....*************** *************

MG06472.1 	EFLERNYKEDMDREQTIRLAIKSLLEVVQTGAKNIEIAIMAPGKSIEMLPVEDIENYVKN
NCU06440.1	EFLERNYKDDMDREATIRLTIKSLLEVVQTGAKNIEIAIMAPGKELEMLPVEDIENYVKN
FG07282.1 	EFLERNYKEDMNREATIRLAIKSLLEVVQTGAKNIEISLMAPGATIETLPTSEIEGYVKE
AN8054.1  	EFLERNHQDDMDREQTIQLTIKSLLEVVQTGAKNIEVAIMSPGKTIEMLPDDQIESYVKS
          	******:::**:** **:*:****************:::*:**  :* ** .:**.***.

MG06472.1 	IEQEKQEEAAKKKTGRTPGTGSAAILTREAGSSDDQ---
NCU06440.1	IEQEKQEEADKKKKGRTPGTGTAAILTRSQDEPGDH---
FG07282.1 	IEQEKQEEAAKKKTGRTPGTGSAAILTRSQDDSAAE---
AN8054.1  	IETEKQEEAAKKKTR--AGTTTAAILTRSGGGESSEARE
          	** ****** ***.   .** :******. .    .: .
```
